# Effect of *Opuntia ficus-indica* Extract in Pro-Healthy Chicken Patties: Physicochemical Properties and Oxidative Stability

**DOI:** 10.3390/foods13233970

**Published:** 2024-12-09

**Authors:** Leticia A. Gonçalves, José M. Lorenzo, Roberto Bermúdez, Mirian Pateiro, Marco Antonio Trindade

**Affiliations:** 1 School of Animal Science and Food Engineering, Universidade de São Paulo, Av. Duque de Caxias, Pirassununga 13635-900, Sao Paulo, Brazil; goncalves.leticia@usp.br; 2 Centro Tecnológico de la Carne de Galicia, Rúa Galicia N-4, Parque Tecnológico de Galicia, San Cibrao das Viñas, 32900 Ourense, Spain; jmlorenzo@ceteca.net (J.M.L.); robertobermudez@ceteca.net (R.B.); mirianpateiro@ceteca.net (M.P.); 3 Área de Tecnología de los Alimentos, Facultad de Ciencias de Ourense, Universidad de Vigo, 32004 Ourense, Spain

**Keywords:** natural antioxidants, prickly pear, antioxidant activity, improvement of lipid profile, lipid oxidation, cleaner label

## Abstract

*Opuntia ficus-indica* is a subtropical fruit rich in dietary fibers, carotenoids, vitamins, minerals, and polyphenols. To substitute synthetic additives, its extracts could become an interesting proposal to preserve quality while adding desirable characteristics to meat products. This study aimed to develop healthier chicken patties (with a structured animal fat replacer) added with prickly pear extract (PPE). The extract was analyzed for total phenolic content and antioxidant activity (FRAP, ABTS, DPPH, and ORAC). Four chicken patty formulations were manufactured with total replacement of animal fat by sesame oil emulsion: control, erythorbate 500 ppm, PPE 500, and PPE 750 ppm. Proximate composition and fatty acid profile were analyzed on day 1, and pH, color, and lipid oxidation on days 1, 4, 8, 12, and 16. PPE treatments showed lower TBARSs (*p* < 0.05) and greater pigment stability at the end of storage, corroborating its potential to delay oxidation reactions. No significant effects on chemical composition, pH, or fatty acid profile were observed (*p* > 0.05). Unsaturated represented 76.2% of total fatty acids. Therefore, PPE is an effective antioxidant by improving oxidative stability without promoting changes in other properties, besides adding cleaner label approaches and the use of natural ingredients to develop meat products reformulated with unsaturated oils.

## 1. Introduction

Poultry, especially chicken, is the most consumed meat worldwide, and the significant increase in the popularity of ready-to-eat and ready-to-cook chicken over raw meat has stimulated the sector to seek alternatives to manufacture value-added products [1]. For example, burgers have become traditional foodstuffs among consumers because they are practical, easy to prepare, sensorially attractive, and nutritious (high content of proteins, fats, vitamins, and minerals) [2]. Fat content, in addition to providing energy, is important for the sensory characteristics of mouthfeel and juiciness, flavor, odor, texture, and other physicochemical properties of processed meat, which are generally manufactured with animal fat [3,4]. However, the perception of animal fat as being rich in saturated fatty acids and cholesterol has led to the belief that these products should be avoided since excessive intake could pose a threat to human health [5].

Non-communicable diseases are the leading cause of death worldwide, with cardiovascular diseases responsible for the highest number of fatalities [6,7]. Various non-modifiable factors (age, gender, ethnicity, etc.), and modifiable factors, such as alcohol consumption, lack of physical activity, smoking, and poor dietary habits (e.g., high intake of salt and saturated fat), interfere in the progression of such diseases [8]. The World Health Organization recommends that total fat consumption should be less than 30% of total energy intake (TEI), saturated fat consumption should be limited to below 10% of TEI, and trans-fat intake should be restricted to less than 1% of TEI [9].

Over the past decade, several initiatives have been implemented to decrease the intake of foods high in saturated fat, including food labeling schemes, healthy eating promotion campaigns, risk assessment measures, and trade consultations [10]. Additionally, recent societal changes in eating habits have led consumers to a greater awareness that diet may have positive or negative effects on well-being and health [11,12]. To attract these consumers and meet the growing demand for high-quality and healthier meat products, the processed meat sector is focusing on developing reformulation alternatives through the replacement of or reduction in ingredients such as saturated fat, salt, and synthetic additives [13].

Therefore, fat intake, especially saturated fat, is pointed out as a crucial aspect to be improved in meat products. In contrast, the consumption of oils rich in unsaturated fatty acids is part of the dietary guidelines of health-related authorities, considering the global burden of nutrition-related chronic diseases [14]. This would mean possible changes in favor of substitutes for animal fats, which are mostly composed of polyunsaturated fatty acids, such as emulsified oleogels and hydrogels [15]. Although this reformulation is favorable to health, unsaturated fatty acids are more susceptible to oxidation than saturated, requiring the addition of compounds that will play primary roles in controlling lipid peroxidation [16].

Due to the high concentration of fatty acids and their manufacturing process, burgers are highly prone to lipid oxidation, leading to the generation of off-flavors associated with the sensory perception of rancidity, decreasing the nutritional value and the viable storage period, as well as affecting the functionality [17,18]. Therefore, the addition of inhibitory components, known as antioxidants, is essential to delay quality losses during storage. Actually, for a long time, the food industry has incorporated different antioxidants to reduce the oxidation effects, particularly the use of synthetic substances that have their approved use within an acceptable daily intake limit [19]. However, the use of synthetic additives goes against the growing trend of consumers looking for cleaner-label products, so there has been an increasing interest in alternatives from natural sources without undesirable health side effects. Studies have shown that essential oils and plant extracts obtained from herbs, seeds, fruits, and agroindustrial residues can be as or more effective than synthetic antioxidants [20,21,22,23].

*Opuntia ficus-indica*, also known as prickly pear or nopal cactus, is a plant belonging to the *Cactaceae* family, native to the tropical and subtropical regions of the Americas, which is gaining global interest because it thrives in arid conditions unsuitable for other crops [24]. Scientific literature shows that in addition to the use of prickly pear in the diet, it can also be used for healthcare as it is a natural source of dietary fiber and bioactive compounds, and multiple components present in *Opuntia* have shown notable experimental evidence in treating and preventing chronic diseases related to inflammation and oxidative stress, demonstrating antioxidant, anti-inflammatory, and anxiolytic properties [25,26,27,28,29,30]. Despite some differences within the composition of different cactus structures, it is possible to find some similarities in their phytochemical composition, namely a source of dietary fibers, amino acids (especially proline and taurine), vitamins C (ascorbic acid), B1, B2, A, and E (α-tocopherol), and minerals (calcium, potassium, magnesium, iron, and phosphorus) [30,31,32,33]. The most important biocompounds in cactus fruit are usually carotenoids, betalain (betacyanns and betaxanthin pigments), and phenolic compounds (e.g., phenolic acids and flavonoids), all of which have potent antioxidant properties [32,34,35,36,37,38]. Consequently, the potential of prickly pear and its components to develop new food ingredients with health-promoting properties or to serve as a source of extracts for application in the food, pharmaceutical, or cosmetic industries can be harnessed.

According to the literature, prickly pear is widely known for its excellent profile of bioactive compounds that have been traditionally used to treat several health disorders and are considered to possess various therapeutic properties. Some reported applications of *O. ficus-indica* in food matrices, including bread [39,40,41], cookies [42,43,44], gluten-free pasta [45], and cake [46], have mainly focused on adding cactus meal (from the cladodes or fruit parts) directly to the product to increase fiber content or for antioxidant purposes. Additionally, there are few reports on its extracts being applied to meat products for antioxidant purposes, requiring more specific studies on its application.

Meat and meat products are still considered as sources of saturated fat, an important issue to address nowadays, so the ultimate goal of this sector is to replace saturated and trans fats with monounsaturated and polyunsaturated fats. In this context, extracts of the aforementioned fruit could be an interesting and innovative proposal to preserve quality and add desirable characteristics (cleaner label and use of natural ingredients) to reformulated meat products with total replacement of animal fat. This project aimed to develop healthier chicken patties (with a structured emulsion rich in unsaturated fatty acids) added with an extract from *Opuntia ficus-indica*, searching for the antioxidant effect that contributes to the preservation of quality characteristics and maintenance of shelf-life stability.

## 2. Materials and Methods

### 2.1. Plant Material and Extract Preparation

**Figure 1:**
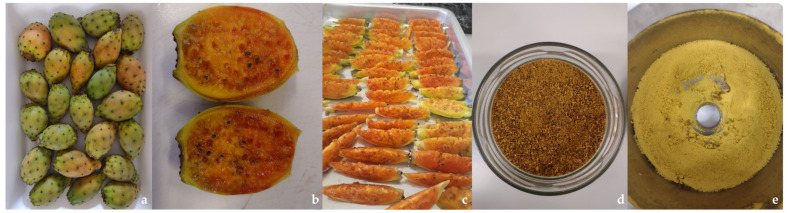
Preparation of prickly pear samples for hydroethanolic extraction and the lyophilized extract obtained. (**a**) Ripe fruits; (**b**) internal part of the fruit; (**c**) sliced fruits (including peel, pulp, and seeds); (**d**) powder obtained after oven drying and grinding; (**e**) extract obtained by hydroethanolic extraction followed by freeze-drying.

The prickly pear fruit (orange variety) was obtained in a single batch from a local market in Campinas (Sao Paulo, Brazil). Ripe fruits (Figure 1a) were picked at the height of the harvest season in Brazil, and processed at the School of Animal Science and Food Engineering (Pirassununga, Brazil) as follows: After they were received, the fruits were selected, washed with running water, sanitized using 70% ethyl alcohol (*v*/*v*), and refrigerated at 4 ± 1 °C in plastic containers until subsequent processes. Then, they were cut lengthwise into slices (Figure 1b,c) and oven-dried for 96 h at 50 °C. The dried material (Figure 1d) was crushed to obtain a fine powder (moisture = 13.22 ± 0.02%; protein = 5.62 ± 0.14%; total fat = 4.20 ± 0.21%; ash = 6.49 ± 0.17%).

The extraction process and the subsequent steps were carried out in the physicochemical laboratory of the Centro Tecnolóxico da Carne (CTC) de Galicia (San Cibrao das Viñas, Spain). The extraction methodology, based on the study of Aruwa et al. [47], consisted of mixing the powder with an 80% ethanol–water solvent (*v*/*v*) at a ratio of 1:10, stirring at 120 rpm for 24 h at room temperature (approximately 24 °C), filtering through Whatman filter paper No. 1, and concentrating in a rotary evaporator (Büchi Labortechnik AG, mod Büchi^®^ Rotavapor R-200, Flawil, Switzerland) under vacuum from 45 to 60 °C. Afterward, the extract was lyophilized (Büchi Lyovapor™ L-300, Flawil, Switzerland) and frozen at −80 °C for later use (Figure 1e). All steps were performed while avoiding exposure to light.

### 2.2. Total Phenolic Content and Antioxidant Activity of the Prickly Pear Extract (PPE)

#### 2.2.1. Determination of the Total Phenolic Content (TPC)

TPC was determined as previously described by Singleton and Rossi [48] with some modifications. Dilutions of PPE (0.5 mL) were mixed with 10% diluted Folin–Ciocalteu reagent (2.5 mL); after 6 min, 7.5% Na_2_CO_3_ solution (2 mL) was added, and the mixture was vortexed. Absorbance (760 nm) was measured after 15 min at 50 °C, and the results were expressed as mg of gallic acid equivalent (GAE)/100 g.

#### 2.2.2. Ferric Reducing/Antioxidant Power (FRAP)

The FRAP assay was conducted according to Benzie and Strain [49], expressing the results as μmol of Fe^2+^/100 g. The reagent was prepared by mixing acetate buffer 0.3 M (pH 3.6), 2,4,6-tripyridyl-s-triazine (TPTZ) 10 mM in 40 mM HCl, and FeCl_3_:6H_2_O 20 mM in a 10:1:1 (*v*:*v*:*v*) ratio. Then, 900 μL of freshly prepared FRAP reagent was reacted with 30 μL of the sample and distilled water (90 μL). After incubation for 20 min at 37 °C, absorbance was measured at 593 nm.

#### 2.2.3. DPPH (2,2-Diphenyl-1-Pcrylhydrazyl) Radical Scavenging Activity

The DPPH^+^ assay was performed with some modifications according to Brand-Williams et al. [50]. Samples (100 μL) were added to 3.9 mL of DPPH solution (60 μM). Absorbance readings (at 515 nm) were obtained after incubation at 37 °C for 10 min. The results were determined using Trolox as a standard and expressed as mg Trolox equivalents (TE)/100 g.

#### 2.2.4. ABTS Radical Cation Decolorization (ABTS) Assay

The assay by Re et al. [51] consists of measuring the reduction in the absorbance of ABTS (2,2-azinobis-(3-ethyl-benzothiazoline-6-sulfonate) solution when reacting with antioxidant compounds. The reagent was prepared by mixing a 7 mM ABTS stock solution with 2.45 mM potassium persulfate. After 12–16 h in darkness, this solution was diluted until an absorbance of 0.700 ± 0.02 and then reacted (980 μL) with the samples (20 μL). The absorbance was measured (734 nm) after 10 min, and the results were expressed as mg of ascorbic acid/100 g.

#### 2.2.5. Oxygen Radical Absorbance Capacity (ORAC) Assay

ORAC was assayed according to Huang et al. [52]. The filters of 485 and 528 nm were used to record fluorescence excitation and emission, respectively, using a BioTek Synergy H1 Hybrid Multimode Reader (Santa Clara, CA, USA). Trolox was used as a standard, and phosphate buffer as blank. The results were calculated based on the net area under the curve, expressed as g Trolox equivalents (TE)/100 g.

### 2.3. Chicken Patty Formulation and Manufacture

To produce a pro-healthy product, four treatments of chicken patties were formulated with the total replacement of traditionally used animal fat with a sesame (*Sesamum indicum*) oil hydrogel emulsion: control (CON; without antioxidant), E500 (commercial sodium erythorbate; 500 mg/kg), and PPE500 and PPE750 (prickly pear extract; 500 and 750 mg/kg, respectively). The sesame oil was emulsified with Prosella^®^ powder, a commercial gelling agent (Laboratorios Amerex S.A., Madrid, Spain). The virgin oil, processed by cold pressing from a commercial local brand, showed the following nutritional allegations: 42% polyunsaturated, 38% monounsaturated, and 14% saturated fatty acids. The guidelines by Barros et al. [53] were used to prepare the emulsion: after mixing water (56 g/100 g) with sesame oil (37.3 g/100 g) at 3000 rpm for 1 min, Prosella^®^ (6.7 g/100 g) was slowly incorporated and homogenized for 3 min and then left at room temperature for 2 h. Finally, the emulsion was refrigerated at 4 °C until needed. To manufacture the patties, the chicken breast, and the oil gel were ground in a refrigerated mincer (La Minerva, mod A/E22R, Bologna, Italy) equipped with a mincing plate of 8 mm and 6 mm in diameter, respectively. The ingredients were homogenized in a mixing machine (Mainca, mod RM-20, Barcelona, Spain) according to the following formulation: white meat (85.8%), oil emulsion (9.0%), salt (1.2%), the antioxidant (erythorbate, PPE or none, depending on the treatment), and cold water. Patties were molded into portions of 100 g each, 10 cm in diameter and 1 cm in height, and packaged in 300 µm thick PET-EVOH-PE trays containing two units per package under a modified atmosphere (70% N_2_; 30% CO_2_). Samples were stored at 2 ± 1 °C under light to simulate supermarket conditions and analyzed in quadruplicate: proximate composition and fatty acid profile on day 1, and pH, color parameters, and lipid oxidation on days 1, 4, 8, 12, and 16 of storage. The whole experiment was repeated in two independent runs. A total of 160 patties were produced for the evaluation of oxidative stability and physicochemical analysis (4 treatments × 5 sampling points × 4 sample repetitions for each sampling point × 2 different processing batches).

### 2.4. Proximate Composition

The proximate composition of the different treatments was determined according to the methods assigned by the International Standards Organization (ISO): moisture [54], protein [55], and ash content [56]. The Am 5–04 procedure [57] was used to assess the total fat.

### 2.5. Color Parameters and pH

After the samples were exposed to atmospheric air for 30 min, instrumental color was measured using a portable colorimeter (CR-600d, Minolta Co. Ltd., Osaka, Japan) in the CIELAB space (lightness, L*; redness (+)/greenness (−), a*; and yellowness (+)/blueness (−), b*). The device was set to a pulsed xenon arc lamp, 10° viewing angle geometry and 8 mm aperture. The total color variation (ΔE*) was calculated to compare differences between treatments during storage:

∆E* = [(L*_4–16_ − L*_1_)^2^ + (a*_4–16_ − a*_1_)^2^ + (b*_4–16_ − b*_1_)^2^]^1/2^ (1)

The pH was analyzed using a digital pH-meter (Hanna Instruments, mod FC232D, Eibar, Spain) equipped with a penetration glass probe, previously calibrated with pH 4.01 and pH 7.01 buffer solutions.

### 2.6. Lipid Oxidation

To assess the degree of lipid oxidation, the secondary products of the reaction were evaluated during storage by the index of 2-thiobarbituric acid reactive substances (TBARSs), spectrophotometrically at 532 nm as proposed by Vyncke [58], and the results were expressed as milligrams of malonaldehyde (MDA)/kg, calculated from a standard curve with 1,1,3,3-tetraethoxypropane (TEP).

### 2.7. Fatty Acid Composition Analysis

Firstly, total fat was extracted as described by Bligh and Dyer [59] with modifications and then transesterified according to the procedure previously described by Domínguez et al. [60], and fatty acid methyl esters (FAMEs) were analyzed following the chromatographic conditions described by Barros et al. [53]. Separation and quantification of FAMEs were carried out using a gas chromatograph, model GC-Agilent 7890B (Agilent Technologies, Santa Clara, CA, USA), equipped with a flame ionization detector (FID) and PAL RTC-120 autosampler, and using a DB-23 fused silica capillary column (60 m, 0.25 mm i.d., 0.25 μm film thickness; Agilent Technologies). The injector and detector were maintained at 260 °C and 280 °C, respectively, with 64.2 mL/min of total flow, and one microliter of the sample was injected in split mode (1:50). The total time for chromatographic analysis was 30 min. Data acquisition and equipment control were performed by Mass Hunter GC/MS Acquisition B.07.05.2479 software (Agilent Technologies, Santa Clara, CA, USA), while data analysis was carried out with the Mass Hunter Quantitative Analysis B.07.01 software.

The retention times of authenticated standards (FAME Mix-37 components, docosapentaenoic acid (C22:5n-3), trans-11 vaccenic acid (11t-C18:1), cis-vaccenic acid (18:1n-7); Supelco, Madrid, Spain) were used to identify individual FAMEs by comparison, and the content was expressed as g/100 g of total fatty acids identified. The following groups of fatty acids were calculated by summing the individual fatty acids: saturated (SFA), monounsaturated (MUFA), and polyunsaturated (PUFA), as well as the PUFA/SFA ratio; in addition, n-6 and n-3 fatty acids and their ratio (n-6/n-3) were calculated to evaluate the nutritional quality of the lipids.

### 2.8. Statistical Analysis

The experiment was performed according to a completely randomized design. A total of 160 chicken patties were analyzed for the different dependent variables (4 treatments × 5 sampling points × 4 sample repetitions for each sampling point × 2 different processing batches). Data were subjected to the two-way analysis of variance (ANOVA) for stability analysis, considering the storage time and treatment as fixed effects, and manufacture replicates were taken into account as a random effect. One-way ANOVA was performed to analyze the variables assessed only on day 1 (proximate composition and fatty acid profile). The difference between averages was assessed using Tukey’s post hoc test when ANOVA was significant (*p* < 0.05). Statistica™ version 13.5.0.17 software (TIBCO Software Inc., Santa Clara, CA, USA) was used to perform statistical analysis.

## 3. Results

### 3.1. Total Phenolic Content and Antioxidant Activity of the Prickly Pear Extract (PPE)

The antioxidant characteristics of prickly pear were estimated using different in vitro methodologies (total phenolic content, FRAP, DPPH, ABTS, and ORAC) to provide an indication of the antioxidant potential of the bioactive compounds present in PPE. These methodologies were chosen because they are rapid and stable methods widely used as significant indicators of antioxidant activity [61], and some authors recommend using at least two of them combined because varying their mechanisms of antioxidant action can provide reliable information [62]. The results can be seen in Table 1.

The Folin–Ciocalteu method used to determine the total phenolic content (TPC) can be considered an electron transfer assay, as it measures the reducing capacity of the sample [63]. The TPC of the prickly pear extract (PPE) was 1438.74 ± 4.43 mg of GAE/100 g in this study (orange variety), which is higher than those obtained from cactus pear pulp of both purple and orange varieties (89.2 and 69.8 mg of GAE/100 g, respectively) [64], the aqueous extract made from the pulp of *Mandarina* (yellow-orange) prickly pear (209.13 mg GAE/100 g) [35], or aqueous extracts obtained from freeze-dried and oven-dried *O. ficus-indica* pulp (425.0 and 432.0 mg of GAE/100 g, respectively) [47], and also higher than similar fruit matrices such as red pitaya (268.13 mg of GAE/100 g), as reported by Belluci et al. [65]. Freeze-dried pulps of the orange- and red-skinned varieties of *O. ficus-indica* presented 1902.0 and 1522.0 mg GAE/100 g, respectively, closer to the results found in this study [66].

Polyphenols are secondary metabolites that plants synthesize in response to stress during their development [67]. They contribute to the antioxidant activity of extracts because they can transfer hydrogen atoms or electrons, blocking oxidative processes caused by the generation of free radicals [68]. In this sense, an increase in polyphenolic compounds entails greater antioxidant properties. Also, antioxidant activity is affected by synergistic and antagonistic interactions of the compounds present in the matrix and the extraction, as well as by the types of phenol and the site and number of hydroxyl groups present in the extract: a better antioxidant power is generated with a greater number of hydroxyl groups [68]. Variations in phenolic content may be affected by many factors, such as fruit maturity, regional climate, solvent used, and differences in processing methods, and hydroalcoholic solvents generally express higher TPC allowed by the extraction of polar and nonpolar compounds [69].

The measurement of the antioxidant activity by FRAP showed 6793.24 ± 142.93 μmol of Fe^2+^/100 g, which indicates a potential to reduce compounds and scavenge free radicals, depending on the ability to donate hydrogen. In other studies that evaluated *Opuntia* varieties, Jorge and Troncoso [70] presented 700.0 μmol Fe^2+^/100 g for aqueous extract of *O. ficus-indica*, while *O. apurimacensis* had 1100.0 μmol Fe^2+^/100 g. Also, regarding the capacity to scavenge free radicals, the DPPH radical assay presented 700.26 ± 30.01 mg TE/100 g, similar to the values presented by Sáenz et al. [71] (750.9 mg TE/100 g for cactus pear pulp and 719.2 mg TE/100 g for the ethanolic extract) and higher than those found by Corral-Aguayo et al. [72] for the acetone/water/acetic acid extract of prickly pear (45.0 mg TE/100 g). Smeriglio et al. [73] applied the DPPH^+^ assay to three different varieties: the values obtained for pulp were 77.15, 100.35, and 55.09 mg TE/100 g (red, orange, and yellow varieties, respectively), and those for the peel were 254.92, 517.11, and 213.77 mg TE/100 g for red, orange, and yellow varieties, respectively.

The ABTS assay is frequently used to measure the antiradical capacity, since it indicates the ability of matrices to trap free radicals by donating hydrogen atoms or electrons, resulting in the bleaching of the colored radical solution [74]. This technique is considered simple and versatile, allowing for the measurement of the antioxidant activity of highly sensitive compounds, which is appropriate for natural compounds. For the ABTS assay, an amount of 1398.21 ± 16.34 mg of ascorbic acid/100 g was found in PPE. De Carvalho et al. [75] reported higher antioxidant activity for turmeric root extract obtained by supercritical fluid extraction (1490.53 mg of ascorbic acid/100 g). A comparison of this result with those found by other studies, as well as the results of some other analyses of antioxidant capacity, was extremely difficult since different standards were used to estimate the antioxidant activity, in addition to the results being represented in different units.

One of the most useful and sensitive methods for evaluating antioxidant activity is the Oxygen Radical Absorbance Capacity (ORAC), which takes into account both reactivity and stoichiometry parameters and measures the hydrophilic antioxidant capacity towards peroxyl radicals by a hydrogen atom transfer reaction mechanism [62,76]. The degree of free radical damage is indicated by the change in the fluorescence intensity index, caused by the degradation of fluorescein by the activity of 2,20-azobis(2-amidinopropane) dihydrochloride (APPH) radicals, and the presence of different classes of antioxidant substances can delay the level of free radical generation [76]. For ORAC, the value of 3.43 ± 0.01 g TE/100 g was obtained. Lower antioxidant activity was found by Albano et al. [64] for the pulp extracts of two varieties of cactus pear using a mixture of ethanol, formic acid, and water as solvents (0.32 and 0.25 g TE/100 g for purple and orange varieties, respectively). Smeriglio et al. [73] applied the ORAC assay to three varieties: the values obtained for the pulp were 0.24, 0.24, and 0.21 g TE/100 g (red, orange, and yellow varieties, respectively), and for the peel, similar values compared to those found in this study, were found, namely 3.4, 4.3, and 2.7 g TE/100 g for red, orange, and yellow varieties, respectively.

The different results obtained from different antioxidant methodologies could be related to the different mechanisms of the reactions involved. The variation in antioxidant activity among different plants can be attributed to a combination of genetic factors, biotic and abiotic conditions, soil characteristics, crop conditions, and the specific microclimate in which these species are cultivated, explaining the controversial data in the literature [77,78]. Moreover, variations in antioxidant capacity found in different studies for *Opuntia* are probably due to the differences observed in many factors, such as ecotypes, physiology, variety, fruit maturity, country of origin, growth and crop conditions, part of the plant investigated (varies within cladodes, fruits or prickly pears, peels, seeds, and flowers), and extraction conditions [79,80,81]. In addition, the biological activity and phytochemical compounds can be affected by technological procedures used to extract and quantify these compounds [82]; for example, the solvent polarity, temperature of extraction, drying process, and particle size can strongly affect the effectiveness of the extraction methods [67,69,83].

The antioxidant actions attributed to plant extracts may not be due to only a single bioactive component, but also to the synergistic effect of multiple substances. Several reports have suggested that the antioxidant properties of the cactus pear are mainly due to its content of polyphenolics, pigments (e.g., a mixture of yellow-orange betaxanthin), and ascorbic acid [31,32,64,80]. Polyphenols are reducing agents recognized for their protective effects against pathogenic bacteria and viruses infecting plants, or UV radiance; they offer robust protection against oxidation by scavenging free radicals, donating hydrogens to counterpoise reactive species, or chelating metals that catalyze oxidation [84,85,86]. Betalains, found in both the epidermis and the pulp of the prickly pear, give the fruit its color, ranging from yellow to purple [64,81]. These pigments (by-products of betalamic acid) include betacyanins, which produce red and blue hues, and betaxanthines, which create yellow and orange tones [81]. The yellow-orange indicaxanthin (proline betaxanthin), whose presence in edible plant sources is limited to cactus pear, and some berries, can donate hydrogen atoms, reflecting the multifaceted nature of antioxidants that could influence the good antioxidant performance of prickly pear orange varieties [64,87]. Betalains are considered cationized antioxidants that present strong protection against free radicals, particularly in biological systems and animal products, as protecting fats from oxidation promotes stability in terms of photodegradation [88,89]. However, not all sources of betalains show the same antioxidant capacity due to the number and position of hydroxyl/imino groups and the differences in the aglycone glycosylation of their structure [90]. As a primary antioxidant, ascorbic acid (vitamin C) has great effects against oxidative-stress-induced cellular damage by neutralizing reactive oxygen species and protecting proteins from alkylation by lipid peroxidation products [91].

All of these compounds are valuable for improving oxidative stability, demonstrating not only fascinating properties for health promotion but also a good candidate as a natural ingredient with applications ranging from food preservation to cosmetics and pharmaceuticals. Due to the high antioxidant potential and high phenolic compound content of PPE, the authors designed a reformulation of meat products with different percentages of PPE to evaluate whether its antioxidant activity is appropriate for protection against lipid oxidation.

### 3.2. Proximate Composition of Chicken Patties

As expected, the proximate composition of chicken patties was not affected by the addition of antioxidants (*p* > 0.05), presenting averages in the range of 74.25–74.12% for moisture, 18.66–18.52% for proteins, 3.41–3.29% for total lipids, and 2.40–2.36% for ash content (Table 2). These results are in agreement with those presented in other studies that replaced animal fat entirely with an emulsion of healthy oils in meat products and adopted the strategy of substituting synthetic antioxidants with natural compounds, such as pork patties produced with the incorporation of red pitaya extract and tiger nut oil emulsion [65], and lamb patties formulated with chia oil emulsion using two different extracts, from guarana seed and pitanga leaf [92].

### 3.3. Color Parameters and pH

**Figure 2:**
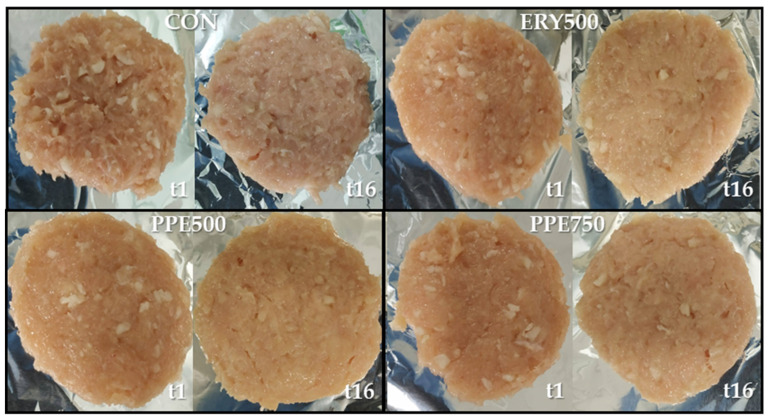
Chicken patties at the beginning (t1) and end (t16) of storage. Treatments: CON: patties prepared without antioxidant; ERY500: patties prepared with sodium erythorbate at 500 mg·kg^−1^; PPE500: patties prepared with prickly pear extract at 500 mg·kg^−1^; PPE750: patties prepared with prickly pear extract at 750 mg·kg^−1^.

The patty samples at the beginning (t = 1 day) and end (t = 16 days) of storage are shown in Figure 2. The color parameters evaluated during the 16 days of refrigerated storage are listed in Table 3. For lightness (L*), a significant difference (*p* < 0.05) was observed between the formulations only on day 1, being higher for PPE750. However, L* did not seem to follow any specific trend over time, despite an increase in ERY500 observed from day 1 to day 16 (*p* < 0.05) as well as the maintenance of values when comparing the beginning and end of storage for the CON, PPE500, and PPE750 treatments.

The different patty formulations did not lead to significant redness (a*) differences (*p* > 0.05) when comparing the same storage time, the same as previously reported by Agregán et al. [93]. Only the control treatment showed a reduction in redness parameters on the surface of chicken patties during storage, while the other three treatments presented similar trends: no significant difference (*p* > 0.05) was observed between days 1 and 16. The results indicate that both natural and synthetic antioxidants protected the redness. Except on day 12, yellowness (b*) parameters were affected by the use of antioxidants, but with no definite trend, although CON tended to have the lowest values at all sampling points. Finally, b* was maintained for all four treatments during the 16 days of refrigerated storage, decreasing only in the middle of the period for the CON treatment and then recovering in the last few days.

In general, ΔE* values did not differ between the treatments when compared to the same period, except at the end of storage (from day 1 to day 16), as the samples with sodium erythorbate possessed the highest color differences. Additionally, the ΔE* values showed significant differences (*p* < 0.05) within the storage time analyzed for three treatments (CON, ERY500, and PPE500), excluding PPE750, which showed that the highest concentration of natural extract applied (750 ppm) was able to maintain the parameter of total color difference during all storage periods. Oxidation processes, such as lipid oxidation, generate pro-oxidants that react with oxymyoglobin, resulting in a buildup of metmyoglobin, a marker of protein oxidation, over time [94]. Antioxidants can help stabilize color by chelating transition metals involved in free radical formation and scavenging these radicals [95]. This process slows down the oxidative reactions that cause discoloration in meat products.

These results are in line with those found by other authors, who noticed a correlation between increasing natural antioxidant concentration and decreasing ΔE* [96]. Furthermore, although PPE500 varied over storage time, the total color difference was not significant when comparing the first (1–4 days) and last (1–16 days) periods investigated. These data may indicate the greater pigment oxidative stability of treatments with *O. ficus-indica* extracts compared to CON and ERY500, which may represent an advantage over other treatments since the change in product color is not desired by consumers.

The pH of chicken patties (Table 3) was not affected by the storage time or different formulations (*p* > 0.05), indicating that the use of antioxidants did not influence the acidity of the samples, maintaining values close to 5.7–5.9 along the sampling points. Similar results were observed in chicken patties formulated with rosemary and green tea extracts [97].

### 3.4. Lipid Oxidation

**Figure 3:**
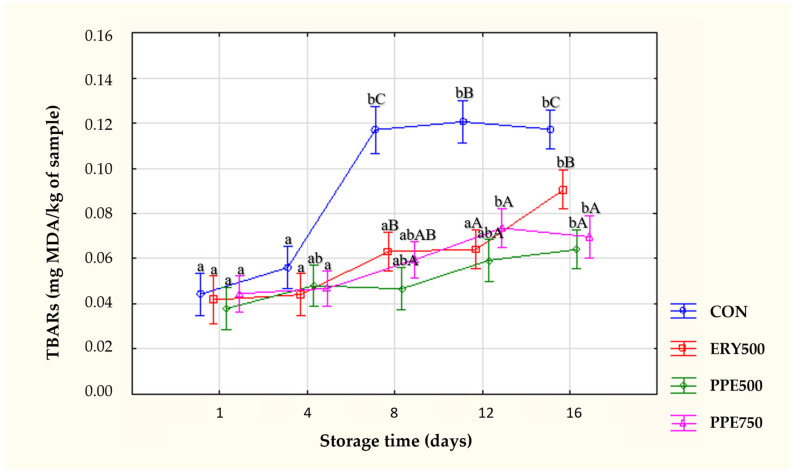
Evaluation of TBARS values (mg MDA/kg of sample) in chicken patties during refrigerated storage. ^a,b^ Mean values horizontally (same treatment in different days) with different letters indicate a significant difference (*p* < 0.05; Tukey’s test); ^A–C^ mean values vertically (different treatment in the same day) with different letters indicate a significant difference (*p* < 0.05; Tukey’s test); Sig.: significance; n.s.: not significant (*p* > 0.05). Treatments: CON: patties prepared without antioxidant; ERY500: patties prepared with sodium erythorbate at 500 mg·kg^−1^; PPE500: patties prepared with prickly pear extract at 500 mg·kg^−1^; PPE750: patties prepared with prickly pear extract at 750 mg·kg^−1^.

The formation of TBARSs (thiobarbituric acid reactive substances) is a common issue in the storage of most processed foods, leading to a significant factor contributing to reduced storage stability. Therefore, lipid stability is one of the most important aspects to consider in the development of meat and meat products, as it affects other quality factors such as protein oxidation and product discoloration during their shelf life [95]. Lipid oxidation (TBARS) mean values are shown in Figure 3. At the beginning of sampling points (days 1 and 4), there were no significant differences (*p* > 0.05) between treatments, but over the time evaluated, the treatment formulated without adding antioxidants (CON) showed the most noticeable increase in the degree of lipid oxidation.

All test samples showed a significant (*p* < 0.05) and gradual increase in TBARS values as storage days progressed, and the control sample exhibited the highest TBARS values, likely due to the absence of any antioxidants. This suggests that while the presence of antioxidants slowed down the rate of lipid oxidation, it could not be completely prevented, and even under refrigerated storage conditions, a gradual increase in TBARS values was observed. However, it should be noted that the indices throughout the storage period were lower than the level considered the limiting threshold for consumers to perceive rancidity, and some authors have established that at around 2.0 mg MDA/kg, the sample can be still considered acceptable for human consumption [98]. On day 16, the highest value was obtained from CON, followed by ERY500, and then PPE750 and PPE500, both of which did not differ significantly (*p* > 0.05), supporting that the prickly pear extract demonstrated antioxidant potential promoted by bioactive compounds capable of scavenging radicals and delaying lipid oxidation processes.

The results of this study are consistent with those of other researchers who investigated the oxidative stability of beef burgers produced with partial replacement of animal fat (pork back fat) by an oil enriched with n-3 fatty acids (canola oil). The authors found that adding freeze-dried pulp from red and orange prickly pear to beef burgers increased oxidative stability during 15 days of refrigerated storage [66]. Compared to control samples (without any antioxidants), the inclusion of 3 and 5% cactus cladode powder in the formulation of beef and chicken burgers proved to be beneficial in decreasing oxidation reactions, protecting meat lipids against oxidation in both raw and grilled burgers analyzed during frozen storage for 100 days [99]. Among emulsified meat products, the incorporation of cactus pear peel flour in cooked sausages decreased oxidative rancidity during storage [100]. These results revealed that bioactive components present in *Opuntia ficus-indica* (cladodes, whole fruit, pulp, and peels), like phenolic acids and flavonoid compounds, can offer a protective effect against lipid oxidation processes [99].

Santos et al. [101] observed that pumpkin flower extracts prevented the evolution of lipid oxidation reactions in raw and cooked chicken patties. The supplementation of chicken nuggets with Basil (*Ocimum basilicum* L.) significantly decreased TBARS content during shelf life [21]. The incorporation of 1% different fruit peel powders (lemon, orange, and grapefruit) delayed the formation of malondialdehyde and lipid peroxides in frozen chicken patties [102]. Grape antioxidant dietary fiber at a concentration of 2% was efficient in maintaining a retarded oxidation process in cooked chicken patties [103], and Basanta et al. [104] reinforced the use of fruit extracts as natural antioxidants, since raw chicken patties supplemented with microparticles of peel and pulp of plum showed 50% lower TBARS values compared to the control treatment (without antioxidant). Therefore, in agreement with the results of the present study, other authors have suggested the use of natural extracts obtained from different plant sources as substitutes for synthetic additives, as they have demonstrated similar or greater efficacy in inhibiting lipid oxidation in meat products.

### 3.5. Fatty Acid Composition of Chicken Patties

Not surprisingly, the fatty acid profile analyzed on day 1 (Table 4) did not differ among the different formulations (*p* > 0.05). This is because the same animal fat substitute was used for all four chicken patty treatments, and the addition of antioxidants did not modify the lipid profile composition at the beginning of storage. PUFAs represented approximately 33.7% of total fatty acids, while MUFAs and SFAs represented 42.5 and 23.8%, respectively. These results revealed that chicken patties made with sesame oil gel emulsions are a potentially viable source of MUFAs and PUFAs, and the fatty acids with the highest concentrations in the samples were oleic acid (C18:1n-9) and linoleic acid (C18:2n-6). Owing to the fatty acid composition, patties enriched with polyunsaturated fatty acids might be more susceptible to oxidative processes than those made with animal fat, such as pork backfat. However, the inclusion of high amounts of unsaturated oils in the diet could provide more benefits for cardiovascular health [105].

The n-6/n-3 ratio is an indicator of the nutritional quality of lipids. The WHO/FAO report indicated that a balanced intake of n-6 and n-3 PUFAs is essential for human health, although the authors have not established a specific recommendation for the maximum n-6/n-3 ratio, but reinforced that these classes of fatty acids have anti-inflammatory properties with a protective effect against atherogenic changes in vascular endothelial cells [106,107].

## 4. Conclusions

The extract obtained from *Opuntia ficus-indica* presented a high total phenolic content and antioxidant activity compared to similar matrices. The manufacture of chicken patties with total replacement of animal fat with a structured oil emulsion resulted in enrichment with unsaturated fatty acids (76.2%), whereas the addition of PPE delayed lipid oxidation compared to treatments formulated without antioxidants or with sodium erythorbate. Greater pigment oxidative stability was obtained for PPE treatments, representing an advantage because changes in product color are not desired by consumers, and no significant effect was noted on chemical composition, pH, or fatty acid profile. Therefore, prickly pear extract has shown effectiveness as a natural antioxidant by maintaining oxidative stability without promoting changes in other product characteristics, demonstrating that it is a promising natural ingredient for the development of healthier and cleaner-labeled meat products to attract 21st-century consumer interest in foods with enhanced nutritional quality.

## Figures and Tables

**Table 1:** Total phenolic content and antioxidant activity of prickly pear extract (PPE).

Analysis	Result for PPE
Total phenolic content (TPC)	1438.74 ± 4.43 mg of GAE/100 g
Ferric reducing/antioxidant power (FRAP)	6793.24 ± 142.93 μmol of Fe^2+^/100 g
DPPH radical scavenging activity	700.26 ± 30.01 mg Trolox equivalents/100 g
ABTS radical cation decolorization	1398.21 ± 16.34 mg of ascorbic acid/100 g
Oxygen Radical Absorbance Capacity (ORAC)	3.43 ± 0.01 g Trolox equivalents/100 g

**Table 2:** Proximate composition of chicken patties.

Component (g/100 g)	Treatment				Sig.
	CON	ERY500	PPE500	PPE750	
Moisture	74.25 ± 0.18	74.24 ± 0.20	74.17 ± 0.26	74.12 ± 0.17	n.s.
Protein	18.52 ± 0.19	18.61 ± 0.13	18.62 ± 0.35	18.66 ± 0.20	n.s.
Fat	3.41 ± 0.48	3.38 ± 0.53	3.41 ± 0.81	3.29 ± 0.32	n.s.
Ash	2.36 ± 0.05	2.40 ± 0.05	2.37 ± 0.05	2.38 ± 0.04	n.s.

Sig.: significance: n.s.: no significant difference (*p* < 0.05). Treatments: CON: patties prepared without antioxidant; ERY500: patties prepared with erythorbate at 500 mg kg^−1^; PPE500: patties prepared with prickly pear extract at 500 mg kg^−1^; PPE750: patties prepared with prickly pear extract at 750 mg kg^−1^.

**Table 3:** Evaluation of pH and color parameters of chicken patties during refrigerated storage.

Parameter	Storage Time (Days)	Treatment				Sig.
		CON	ERY500	PPE500	PPE750	
L*	1	61.16 ± 3.08 ^abAB^	58.90 ± 0.74 ^aA^	60.30 ± 1.20 ^abAB^	62.15 ± 1.0 5 ^bABC^	*
	4	60.03 ± 3.06 ^A^	59.09 ± 2.80 ^A^	59.70 ± 2.62 ^A^	58.73 ± 1.90 ^A^	n.s.
	8	62.05 ± 2.75 ^AB^	62.17 ± 1.89 ^A^	60.71 ± 2.67 ^AB^	62.42 ± 1.86 ^ABC^	n.s.
	12	62.00 ± 4.26 ^AB^	60.13 ± 2.40 ^A^	62.60 ± 1.59 ^AB^	63.88 ± 2.23 ^BC^	n.s.
	16	65.97 ± 2.64 ^B^	66.78 ± 2.68 ^B^	64.24 ± 1.82 ^B^	65.89 ± 1.32 ^C^	n.s.
	Sig.	*	*	*	*	
a*	1	1.58 ± 0.22 ^B^	1.39 ± 0.30	1.44 ± 0.15	1.59 ± 0.18	n.s.
	4	1.37 ± 0.22 ^B^	1.61 ± 0.58	1.67 ± 0.78	1.71 ± 0.70	n.s.
	8	1.25 ± 0.07 ^AB^	1.47 ± 0.38	1.59 ± 0.15	1.69 ± 0.36	n.s.
	12	1.02 ± 0.44 ^AB^	1.18 ± 0.40	1.34 ± 0.43	1.24 ± 0.42	n.s.
	16	0.79 ± 0.27 ^A^	1.09 ± 0.26	1.14 ± 0.28	1.25 ± 0.41	n.s.
	Sig.	*	n.s.	n.s.	n.s.	
b*	1	15.62 ± 0.72 ^bB^	14.30 ± 0.51 ^a^	15.59 ± 0.86 ^b^	16.41 ± 0.70 ^b^	*
	4	13.39 ± 0.93 ^aA^	14.32 ± 0.63 ^ab^	14.84 ± 1.36 ^ab^	14.95 ± 1.43 ^b^	*
	8	13.42 ± 0.72 ^aA^	13.56 ± 0.56 ^ab^	14.03 ± 0.60 ^ab^	14.49 ± 1.02 ^b^	*
	12	14.99 ± 2.41 ^AB^	14.88 ± 1.85	15.72 ± 1.32	15.27 ± 1.58	n.s.
	16	14.20 ± 1.08 ^aAB^	14.83 ± 1.23 ^ab^	14.12 ± 0.95 ^a^	15.81 ± 0.94 ^b^	*
	Sig.	*	n.s.	n.s.	n.s.	
ΔE*	1–4	2.90 ± 0.68 ^A^	2.86 ± 0.53 ^A^	3.31 ± 0.65 ^AB^	3.06 ± 0.99	n.s.
	1–8	4.19 ± 0.72 ^A^	3.31 ± 0.99 ^A^	3.03 ± 0.93 ^A^	3.10 ± 0.46	n.s.
	1–12	3.12 ± 0.67 ^A^	3.10 ± 0.54 ^A^	3.05 ± 1.01 ^A^	3.56 ± 0.56	n.s.
	1–16	5.97 ± 1.46 ^aB^	8.14 ± 0.75 ^bB^	4.68 ± 0.79 ^aB^	4.66 ± 0.72 ^a^	*
	Sig.	*	*	*	n.s.	
pH	1	5.84 ± 0.04	5.87 ± 0.03	5.86 ± 0.01	5.86 ± 0.01	n.s.
	4	5.88 ± 0.03	5.92 ± 0.03	5.91 ± 0.03	5.91 ± 0.02	n.s.
	8	5.74 ± 0.09	5.76 ± 0.08	5.76 ± 0.06	5.76 ± 0.08	n.s.
	12	5.79 ± 0.07	5.83 ± 0.06	5.83 ± 0.08	5.82 ± 0.07	n.s.
	16	5.76 ± 0.05	5.75 ± 0.07	5.74 ± 0.10	5.73 ± 0.10	n.s.
	Sig.	n.s.	n.s.	n.s.	n.s.	

^a,b^ Mean values in the same row (different treatment in the same day) with different letters indicate a significant difference (*p* < 0.05; Tukey’s test); ^A–C^ mean values in the same column (same treatment in different days) with different letters indicate a significant difference (*p* < 0.05; Tukey’s test); Sig.: significance; n.s.: not significant (*p* > 0.05). Treatments: CON: patties prepared without antioxidant; ERY500: patties prepared with sodium erythorbate at 500 mg·kg^−1^; PPE500: patties prepared with prickly pear extract at 500 mg·kg^−1^; PPE750: patties prepared with prickly pear extract at 750 mg·kg^−1^. * significant different at *p* < 0.05.

**Table 4:** Main fatty acids in the composition of chicken patties.

Fatty Acid	Treatment				Sig.
	CON	ERY500	PPE500	PPE750	
C16:0	15.89 ± 0.98	15.90 ± 0.96	15.87 ± 1.17	15.79 ± 1.21	n.s.
C18:0	6.34 ± 0.13	6.33 ± 0.08	6.35 ± 0.09	6.35 ± 0.07	n.s.
SFAs	23.82 ± 0.97	23.84 ± 1.02	23.81 ± 1.21	23.72 ± 1.32	n.s.
C16:1n-7	2.41 ± 0.52	2.42 ± 0.50	2.40 ± 0.61	2.37 ± 0.62	n.s.
C18:1n-9	37.54 ± 0.11	37.54 ± 0.06	37.53 ± 0.11	37.58 ± 0.06	n.s.
C18:1n-7	2.03 ± 0.07	2.04 ± 0.04	2.04 ± 0.07	2.02 ± 0.10	n.s.
MUFAs	42.49 ± 0.62	42.52 ± 0.63	42.48 ± 0.80	42.47 ± 0.81	n.s.
C18:2n-6	31.50 ± 1.52	31.44 ± 1.59	31.52 ± 1.93	31.63 ± 2.10	n.s.
n-6	32.67 ± 1.59	32.61 ± 1.65	32.69 ± 2.02	32.78 ± 2.16	n.s.
C18:3n-3	0.59 ± 0.03	0.58 ± 0.02	0.58 ± 0.04	0.58 ± 0.04	n.s.
C20:5n-3 (EPA)	0.11 ± 0.01	0.11 ± 0.01	0.11 ± 0.01	0.11 ± 0.01	n.s.
C22:5n-3 (DPA)	0.22 ± 0.01	0.23 ± 0.02	0.22 ± 0.08	0.23 ± 0.01	n.s.
C22:6n-3 (DHA)	0.08 ± 0.01	0.09 ± 0.01	0.08 ± 0.01	0.08 ± 0.00	n.s.
n-3	1.02 ± 0.02	1.03 ± 0.03	1.02 ± 0.01	1.02 ± 0.04	n.s.
PUFAs	33.69 ± 1.58	33.64 ± 1.64	33.71 ± 2.01	33.80 ± 2.13	n.s.
**Nutritional Indices**					
n-6/n-3	32.03	31.66	32.05	32.13	n.s.
PUFA/SFA	1.41	1.41	1.42	1.42	n.s.

Sig.: significance; n.s.: no significant difference (*p* > 0.05). Treatments: CON: patties prepared without antioxidant; ERY500: patties prepared with sodium erythorbate at 500 mg·kg^−1^; PPE500: patties prepared with prickly pear extract at 500 mg·kg^−1^; PPE750: patties prepared with prickly pear extract at 750 mg·kg^−1^. SFAs: saturated fatty acids; MUFAs: monounsaturated fatty acids; PUFAs: polyunsaturated fatty acids; n-3: omega-3; n-6: omega-6.

## Data Availability

The original contributions presented in the study are included in the article, further inquiries can be directed to the corresponding author.
